# YVO_4_:Nd^3+^ nanophosphors as NIR-to-NIR thermal sensors in wide temperature range

**DOI:** 10.1038/s41598-017-18295-w

**Published:** 2017-12-21

**Authors:** I. E. Kolesnikov, A. A. Kalinichev, M. A. Kurochkin, E. V. Golyeva, E. Yu. Kolesnikov, A. V. Kurochkin, E. Lähderanta, M. D. Mikhailov

**Affiliations:** 1 St. Petersburg State University, 7/9 Universitetskaya nab, 199034 St. Petersburg, Russia; 2 Lappeenranta University of Technology LUT, Skinnarilankatu 34, 53850 Lappeenranta, Finland; 3 Peter the Great St. Petersburg Polytechnic University, St. Petersburg, Russia; 4 Scientific and Technological Institute of Optical Material Science, VNTs S. I. Vavilov State Optical Institute, Babushkina 36–1, 192171 St. Petersburg, Russia; 5 Volga State University of Technology, Lenin sqr. 3, 424000 Yoshkar-Ola, Russia

## Abstract

We report on the potential application of NIR–to–NIR Nd^3+^-doped yttrium vanadate nanoparticles with both emission and excitation operating within biological windows as thermal sensors in 123–873 K temperature range. It was demonstrated that thermal sensing could be based on three temperature dependent luminescence parameters: the luminescence intensity ratio, the spectral line position and the line bandwidth. Advantages and limitations of each sensing parameter as well as thermal sensitivity and thermal uncertainty were calculated and discussed. The influence of Nd^3+^ doping concentration on the sensitivity of luminescent thermometers was also studied.

## Introduction

Measuring temperature at micro- and nanoscale is an important task in science, technology and medicine^1–4^. Last decade the biomedical field becomes the research area in which the nanothermometers have found high impact and applicability since the sizes of the nanoparticles are similar to those of the majority of biological objects (cells, bacterias, etc.). Successful application in this area imposes additional requirements on nanothermometers: they have to combine an operation within physiological temperature range (295−320 K)^1^ with biocompatibility and noninvasiveness, and working in moving biological fluids^5^. Along with biomedicine, nanothermometers can be used in various industrial applications: for example, in photonic, electronic and optoelectronic devices and circuits, that are being continuously miniaturized, and whose temperature strongly affects their performance. It should be noted, that industry application usually requires wider temperature intervals than aforementioned physiological range^6–8^.

Among the diverse approaches to thermal sensing at micro- and nanoscale, fluorescence nanothermometry has attracted significant attention due to noninvasive, contactless, and easy to use, unlike conventional tools, such as thermocouple and fiber optic probes or volumetric methods (e.g., ultrasound, computed tomography, and magnetic resonance thermometry)^9^. Fluorescence sensors should be materials that upon optical excitation emit light and their temperature dependence can be calibrated using the changes of one or more measurable parameters, such as the emission intensity, bandwidth, spectral position, polarization or lifetime of the emitting level^10^. Various luminescent materials such as organic dyes^11,12^, polymer nanoprobes^13,14^, layered double hydroxides^15,16^, quantum dots^17,18^, and rare earth doped inorganic nanoparticles^19–22^ have been proposed as nanothermometers. Unique spectral features (narrow absorption and emission lines, relatively long emission lifetimes) together with good chemical and physical stability make rare earth doped inorganic nanoparticles one of the most promising candidates for thermal sensing in wide temperature range through fluorescence thermometry.

Up to now, the great number of rare earth doped phosphors has been successfully demonstrated as efficient thermometers especially in the biological temperature range. Majority of down- and up-converting fluorescence thermometers doped with Nd^3+^, Gd^3+^, Dy^3+^, Ho^3+^, Er^3+^, Tm^3+^ or Eu^3+^ are based on luminescence intensity ratio, whereas only a few scientific papers are devoted to the thermometers based on other temperature dependent luminescence parameter. Therefore, it is very important to carry out a systematic study of advantages and limitations of various thermal sensing luminescence parameters.

Here, thermal sensing was based on monitoring three different temperature dependent parameters of NIR-to-NIR YVO_4_:Nd^3+^ phosphors: the luminescence intensity ratio, the spectral line position and the line bandwidth. It was found that aforementioned luminescence temperature dependent parameters can provide thermal sensing in wide range (123–873 K), which is sufficient for majority of industry application. The influence of doping concentration and sensing parameter on thermal sensitivity was studied and discussed in detail. The temperature uncertainties of YVO_4_:Nd^3+^ 2.4 at.% nanothermometer based on different sensing parameter were calculated and compared.

## Results and Discussion

Phase composition and size of synthesized YVO_4_:Nd^3+^ nanoparticles (NPs) have been studied in our previous paper^23^. It was found that all samples of concentration series have single tetragonal phase without impurities. Mean size of nanoparticles (68 nm) obtained using static light scattering technique matches well with average size (60–70 nm) observed using scanning electron microscopy.

Commercial 808 nm laser diode can effectively excite emission of YVO_4_:Nd^3+^ NPs. It was found that Nd^3+^ ions in YVO_4_ host have quite broad excitation bandwidths (14 nm), therefore they are practically unaffected by small changes in the excitation wavelength (i.e., in the diode temperature)^20^. Cost effective 808 nm diodes also have an advantage compared with 790 nm ones often used for other Nd^3+^ doped NPs from the biological point of view^24,25^.

All luminescence nanothermometers can be divided into different groups based on the particular parameter of luminescence which is analyzed, and from which parameter the thermal reading is ultimately extracted. Emission intensity, spectral line position, bandwidth, lifetime, and polarization could be used to determine local temperature^2^. One of the most widespread methods is ratiometric approach in which thermal reading is achieved by comparing the relative intensity between different spectral lines. Luminescence intensity ratio (LIR) prevents errors in measurements arising from power fluctuations of the excitation source, variations on the concentration of luminescent nanoparticles, and inhomogeneities of the material^26^. LIR technique constitutes a self-referencing method to compute the absolute temperature, since one spectrum contains all of the information needed, avoiding the use of an internal reference^1^.

**Figure 1:**
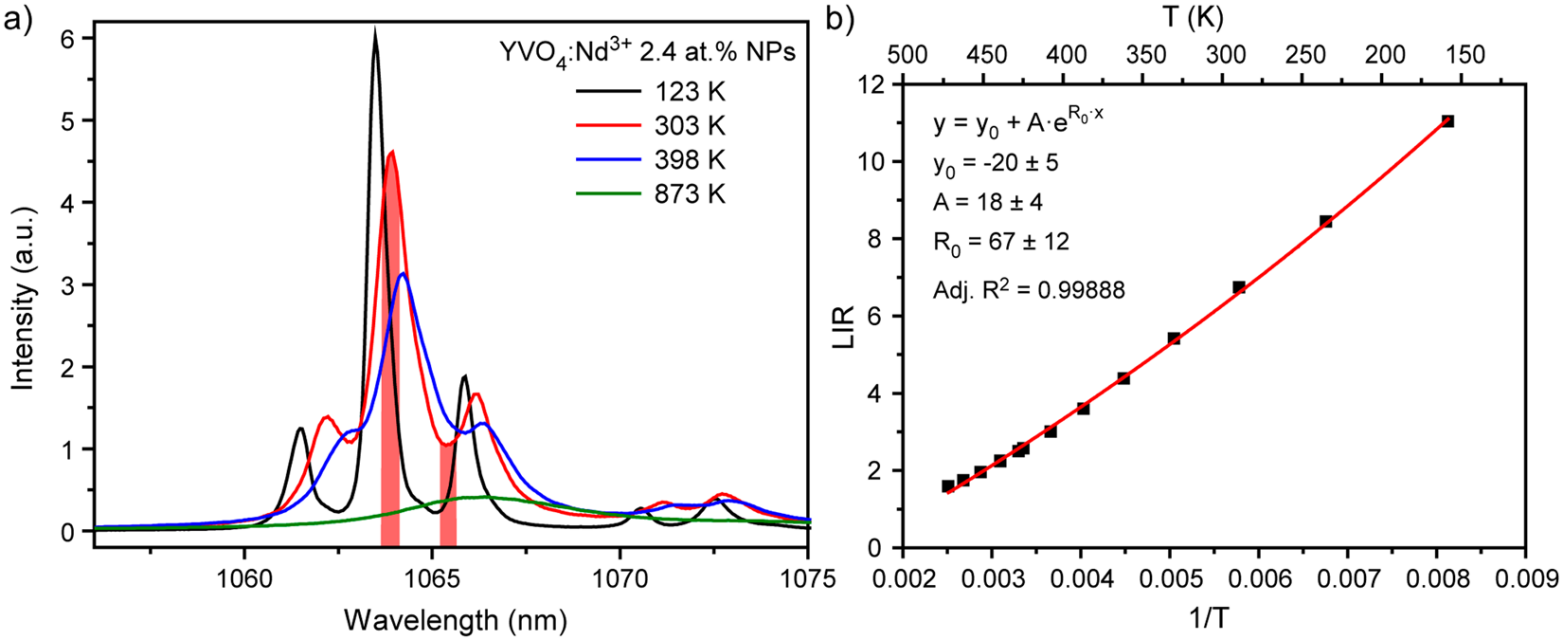
(**a**) Emission spectra of YVO_4_:Nd^3+^ 2.4 at.% NPs obtained at different temperatures; (**b**) evolution of luminescence intensity ratio between peak 1063.9 nm and valley 1065.3 nm (T = 123–398 K).

Nowadays, there are a lot of papers demonstrated successful application of LIR technique in Nd^3+^-doped materials for thermal sensing in the biological temperature range^23,27–29^. LIR was obtained using emission lines lying in the first, second and third biological windows^30–33^. Recently, we demonstrated that thermal sensitivity of nanothermometers could be significantly enhanced by calculation of LIR between emission line (1063.9 nm) and valley (1065.3 nm)^34^. However, quite small energy separation between wavelengths used for LIR calculation limits possible temperature range for sensing. Here, we have defined LIR technique temperature limits for YVO_4_:Nd^3+^ NPs emitting in the second biological window. Figure 1a shows emission spectra generated upon 808 nm excited YVO_4_:Nd^3+^ 2.4 at.% NPs obtained at different temperatures (123, 303, 398 and 873 K). It should be noted that considered temperature region is much broader than usually studied physiological range. As can be seen from Fig. 1a, temperature increase led to the line broadening and shifting. As a result, valley used for LIR calculation almost disappeared at T = 398 K, so thermal sensing based on LIR technique at temperatures which exceed 398 K is impossible. It should be noted that the same result would be obtained for LIR between emission lines 1063.9 nm (^4^F_3/2_(1) − ^4^I_11/2_(1)) and 1071.1 nm (^4^F_3/2_(2) − ^4^I_11/2_(3)) due to the significant broadening of spectral lines at high temperatures.

Temperature induced change of LIR is explained by modification of energy levels population according to the Boltzmann formula:

1 $$LIR \sim A\cdot \exp (-\frac{{\rm{\Delta }}E}{kT})\,$$

where A is a temperature-independent constant, ΔE is the energy gap between energy levels, k is the Boltzmann’s constant and T is the absolute temperature. Despite the fact that equation (1) should be used for LIR between peaks, it was found that the obtained LIR between peak and valley can be also fitted by this formula (Fig. 1b). However, it should be noted, that the fitting parameters had quite big standard errors. Taking into account the obtained results, we can draw a conclusion that the usable temperature range of YVO_4_:Nd^3+^ NPs emitting in the second biological window based on LIR technique is 123–398 K.

In order to broaden the temperature range, other spectroscopic parameters were suggested for thermal sensing. It was found that increase of temperature leads to the red shift of some emission line positions. When the crystal temperature is increased, energy levels and, therefore, spectral lines broaden, invariably as the higher phonon modes are occupied. Since there are many high-lying energy levels which couples, an energy level is normally lowered. Further, it is usually the case that the temperature dependence of higher levels is larger than for lower levels because of smaller energy denominators. As a result, the spectral lines are observed to shift normally to the longer wavelengths when the temperature is increased^35,36^. Thermal shifts to the blue can be caused by thermal expansion of the crystal lattice due to the changes of crystalline-field strength and impurity-level energies. Such shifts were observed for some transitions of Nd^3+^ in the soft hydrated crystal Pr(NO_3_)_3_·6H_2_O. However, it was found that thermal expansion is negligible in hard ionic crystals^35,36^.

**Figure 2:**
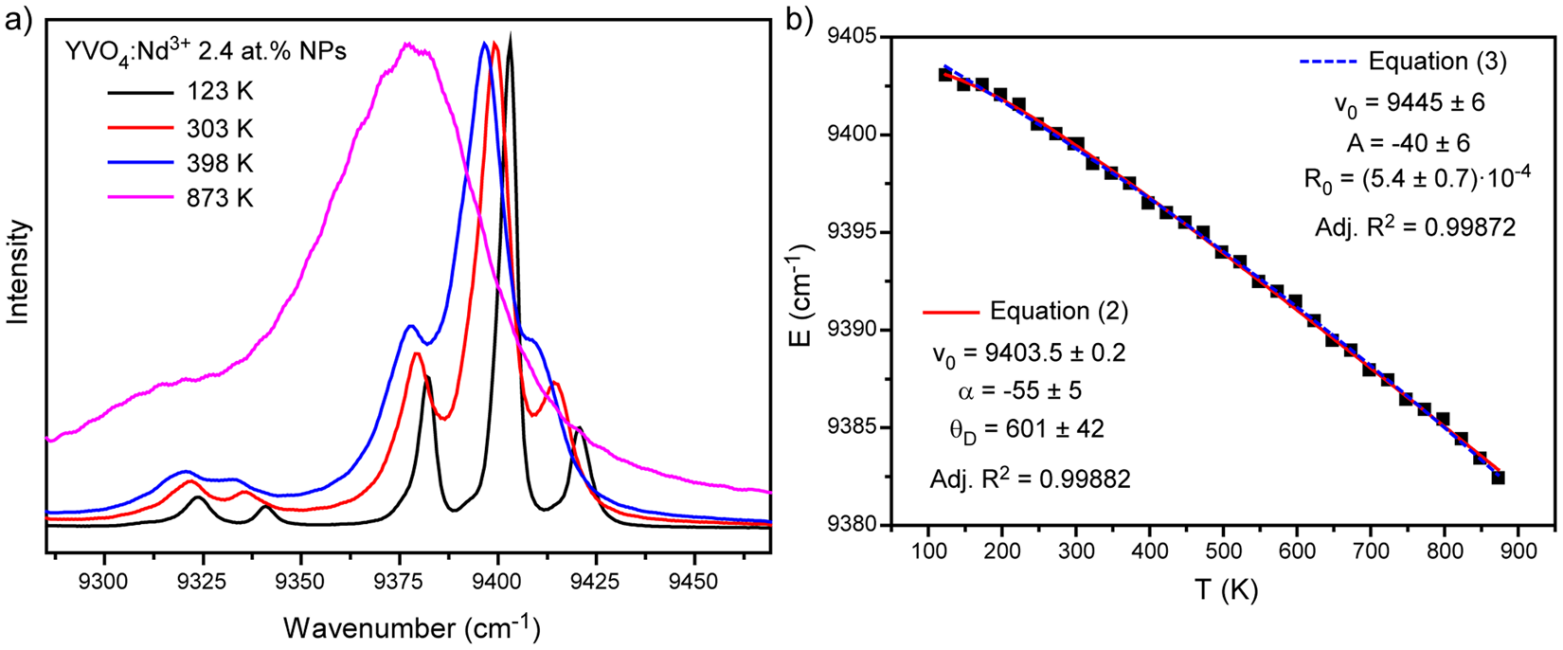
(**a**) Normalized emission spectra of ^4^F_3/2_ − ^4^I_11/2_ transition obtained at different temperatures (123–873 K); (**b**) line position of ^4^F_3/2_ (R_1_) − ^4^I_11/2_ (Y_1_) transition as a function of temperature.

So, shifting of the emission line is usually associated with the electron–phonon coupling effect, which results from the fact that at higher temperatures host vibration modes introduce random perturbation of the ion’s local environment^37^. According to the phonon theory^38,39^, the line position is affected by the crystal strain inhomogeneity, direct one-phonon processes, multiphonon processes, and Raman phonon scattering processes. As was found in earlier studies^36,40^, thermal shift is mainly governed by electron–host interaction effect associated with Raman scattering, and therefore the simplified theoretical expressions for the line shift can be written in the following form^41^:

2 $$\upsilon ={\upsilon }_{0}+\alpha {(\frac{T}{{{\rm{\Theta }}}_{D}})}^{4}{\int }_{0}^{{{\rm{\Theta }}}_{D}/T}\frac{{x}^{3}}{{e}^{x}-1}dx$$

where υ_0_, α, Θ_D_ represent the initial line position (determined at low temperature, in this paper at 123 K), the electron–host coupling parameter, and the effective Debye temperature, respectively. We monitored position of the most intensive emission band of YVO_4_:Nd^3+^ 2.4 at % NPs, which is attributed to the transition between the Stark levels of the ^4^F_3/2_ and ^4^I_11/2_ states (Fig. 2a). Spectral position of aforementioned ^4^F_3/2_ (R_1_) − ^4^I_11/2_ (Y_1_) transition is shown in Fig. 2b. The growth of temperature from 123 K to 873 K causes monotonical red shift of the emission line’s position which can be perfectly fitted by equation (2) (red curve in Fig. 2b). It should be noted that in spite of clear physical meaning, aforementioned function cannot be used for thermal sensing due to the its complexity and inability to derive temperature as a function of spectral shift. Therefore, a simple exponential expression was suggested for fitting the observed line shift as a function of temperature:

3 $$\upsilon ={\upsilon }_{0}+A\cdot {e}^{{R}_{o}T}$$

As can be seen from Fig. 2b, both fitting procedures resulted in similar values of adjusted R^2^: 0.99882 and 0.99872 for equation (2) and (3), respectively. So, proposed fitting function allows using spectral shift of emission line for thermal sensing.

The second consequence of the electron–phonon interaction is a broadening of the emission lines at raised temperatures (Fig. 2a). Linewidth is affected by the same processes which influenced on line position. According to the phonon theory^38^, the width of energy level is given by:

4 $${\rm{\Delta }}\upsilon ={\rm{\Delta }}{\upsilon }^{strain}+{\rm{\Delta }}{\upsilon }^{D}+{\rm{\Delta }}{\upsilon }^{M}+{\rm{\Delta }}{\upsilon }^{R}$$

The first term, ${\triangle \upsilon }^{{strain}}$, is the width due to the crystal strains. The second term, ${\triangle \upsilon }^{D}$, is the width due to direct one-phonon process between the selected energy level and other nearby levels, and consists of a temperature-independent part, which is due to spontaneous one-phonon emission, and a temperature-dependent part. The third term, ${\triangle \upsilon }^{M}$, is the contribution to the width from the multiphonon emission processes, which are temperature independent. The last term, ${\triangle \upsilon }^{R}$ represents the width for the Raman multiphonon process associated with phonon scattering by impurity ions^42^. It should be noted that the first term represents inhomogeneous broadening with a Gaussian line shape due to crystal strains, whereas the other terms give rise to homogeneous broadening with a Lorentzian line shape. Since different line shapes are expected for several broadening mechanisms, simple summation of equation 4 is a rough approximation, and line shape composed of both homogeneous and inhomogeneous parts can be represented by a Voigt profile^43^. If it is necessary, line width may be resolved into homogeneous and inhomogeneous contributions by using the numerical tables prepared by Posener^44^.

It was earlier found that main contribution to line broadening is Raman scattering process which consists of the absorption of one phonon and the emission of another phonon without changing the electronic state of the ion^36,42^. In this case, the width of the energy level can be given by following expression:

5 $${\rm{\Delta }}\upsilon ={\rm{\Delta }}{\upsilon }_{0}+\bar{\alpha }{(\frac{T}{{{\rm{\Theta }}}_{D}})}^{7}{\int }_{0}^{{{\rm{\Theta }}}_{D}/T}\frac{{x}^{6}{e}^{x}}{{({e}^{x}-1)}^{2}}dx$$

where ${\triangle \upsilon }_{0}$ is the initial linewidth, $\bar{\alpha }$ is the coupling coefficient for the electron-phonon interaction and ${{\rm{\Theta }}}_{D}$ is the effective Debye temperature.

**Figure 3:**
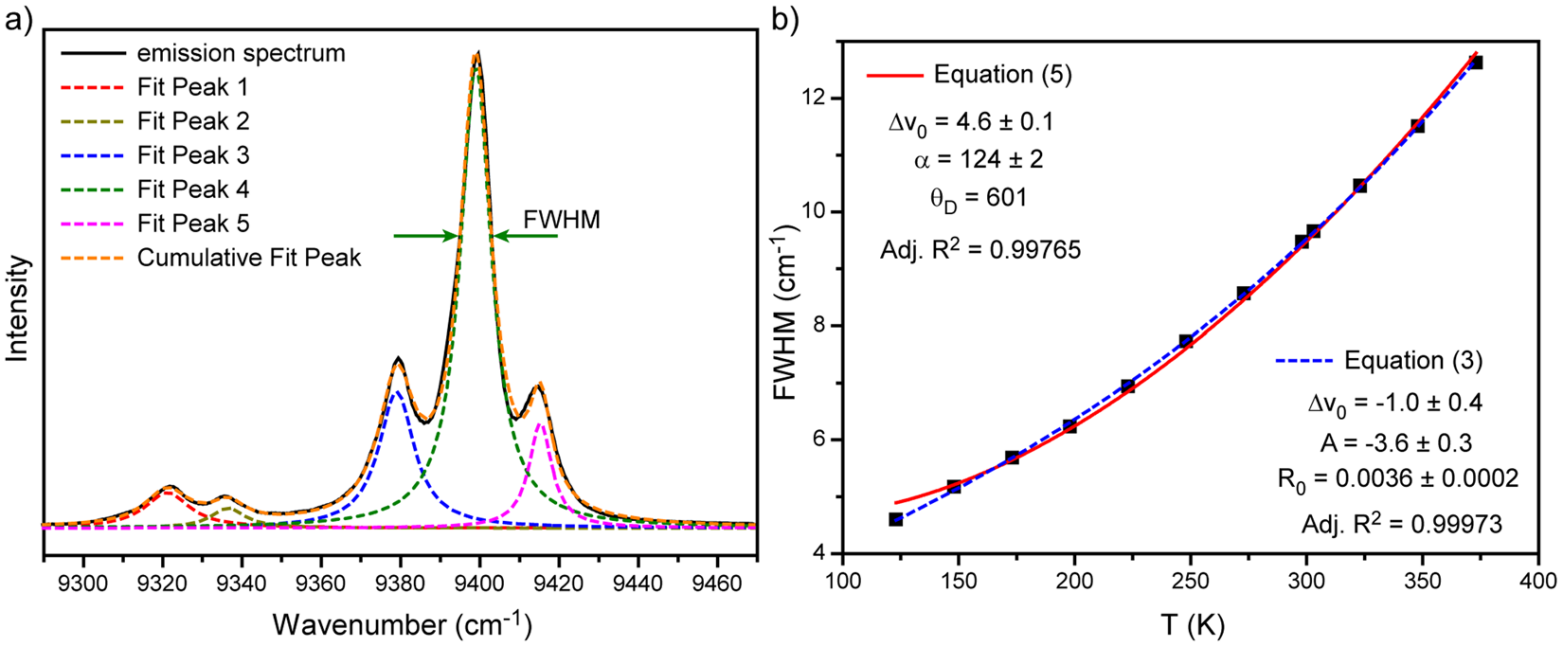
(**a**) Deconvolution of ^4^F_3/2_ (R_i_) − ^4^I_11/2_ (Y_j_) transition; (**b**) FWHM of ^4^F_3/2_ (R_1_) − ^4^I_11/2_ (Y_1_) transition as a function of temperature with fits to equations (5) and (3).

**Figure 4:**
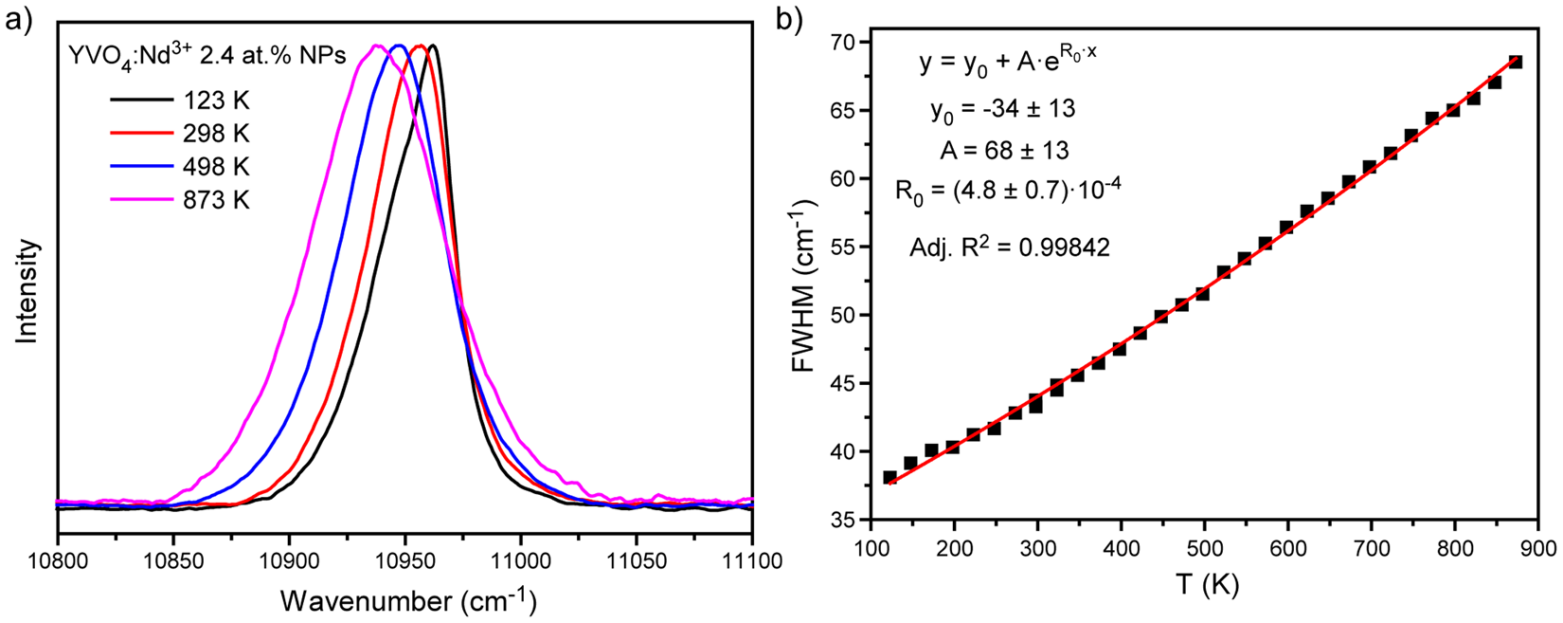
(**a**) Normalized emission spectra of ^4^F_3/2_ (R_1,2_) − ^4^I_9/2_ (Z_5_) transition obtained at different temperatures (123–873 K); (**b**) FWHM of ^4^F_3/2_ (R_1,2_) − ^4^I_9/2_ (Z_5_) transition as a function of temperature with fit to equation (3).

In our case the monitored band is attributed to the most intensive ^4^F_3/2_ (R_1_) − ^4^I_11/2_ (Y_1_) transition (Fig. 3a). The linewidth was obtained from deconvolution procedure. This procedure gave bad results at temperatures higher than 398 K due to the temperature induced line broadening. Figure 3b presented the evolution of linewidth as a function of temperature. The obtained experimental data were fitted by equation (5) with previously defined effective Debye temperature ${{\rm{\Theta }}}_{D}$ = 601 K. The experimental data were also fitted by simple exponential function given by equation (3). However, even simple fitting does not make R_1_ − Y_1_ linewidth a good parameter for thermal sensing because of the proximity of other lines and, as a result, necessity to provide deconvolution procedure. So, we can draw a conclusion that a single emission line should be used for the best thermal sensing when based on line broadening. The emission line centered at about 10956 cm^−1^ (912.5 nm) perfectly fits this requirement (Fig. 4a). Actually, this band consists of two different lines corresponded to the ^4^F_3/2_ (R_2_) − ^4^I_9/2_ (Z_5_) and ^4^F_3/2_ (R_1_) − ^4^I_9/2_ (Z_5_) transitions which can be resolved only at very low temperatures (T ~ 10 K)^42^. It was found that the observed emission line can be well approximated by a Voigt profile in the considered temperature range (123–873 K). The temperature dependence of the full width at half maximum (FWHM) of the emission band is shown in Fig. 4b. The fitting of experimental data was made by the exponential function (equation 3), which confirms simplicity of practical application of such parameter for thermal sensing.

One of the most important parameter for characterizing luminescent thermometers is the relative thermal sensitivity, which is defined as follows^26^:

6 $$S=\frac{1}{\Lambda }\frac{{\rm{\Delta }}\Lambda }{{\rm{\Delta }}T}\,$$

where Λ is the temperature dependent parameter (LIR, Δν or FWHM) and ΔΛ expresses the change in this parameter with change of temperature, ΔT^37^.

The relative thermal sensitivity of all temperature dependent parameters was obtained at T = 303 K (or at T = 298 K in case of FWHM). Example of the relative thermal sensitivity calculation based on LIR is presented below:

7 $${S}_{LIR}=\frac{1}{LI{R}_{303K}}\frac{|LI{R}_{323K}-LI{R}_{303K}|}{{\rm{\Delta }}T=20\,K}$$

Calculation of the relative thermal sensitivity based on other parameters is presented in Supporting Information. It should be emphasized that in case when temperature dependent parameter is the spectral line position, Λ value should be a spectral shift between emission line [in cm^−1^] at T = 303 K and at T = 0 K, unlike the spectral line position at considered temperature as was shown by Ł. Marciniak *et al*.^37^.

Since all three parameters were found to be temperature dependent, each of them was examined as a function of the Nd^3+^ concentration. Here, YVO_4_:Nd^3+^ concentration series consisting of 0.6, 2.4 and 4.8 at.% doped samples were studied. All of the calculated thermal sensitivities for YVO_4_:Nd^3+^ NPs based on the different types of luminescent parameters are listed in Table 1.

**Table 1:** Comparison of sensitivities for different types of Nd^3+^-doped luminescent thermometers.

Material	Sensitivity [% K^−1^]			Ref.
	LIR	Line position	FWHM	
YVO_4_:Nd^3+^ 0.6 at.%	0.51	0.41	0.07	This work
YVO_4_:Nd^3+^ 2.4 at.%	0.54	0.75	0.14	This work
YVO_4_:Nd^3+^ 4.8 at.%	0.47	0.33	0.08	This work
LiLaP_4_O_12_:Nd^3+^ 1%	0.31	0.47	0.32	^37^
LiNdP_4_O_12_	0.003	0.003	0.46	^37^
LaF_3_:Nd^3+^	0.26	—	—	^27^
YAG:Nd^3+^	0.15	—	—	^29^
NaYF_4_:Nd^3+^	0.12	—	—	^45^

The highest thermal sensitivity was found for line position method reaching up to 0.75% K^−1^ for YVO_4_:Nd^3+^ 2.4 at.%, while the lowest was found for FWHM based luminescent thermometers. It should be noted that the thermal sensitivity did not demonstrate monotonical dependence on Nd^3+^ doping concentration: the best performance was achieved for 2.4 at.% doped sample for all sensing techniques. The perspective candidate for a non-contact temperature sensor should possess not only the high thermal sensitivity but also the simplicity of the readout. It is important to note that accurate determination of line position or FWHM are quite difficult in real experiments due to the necessity of providing high resolution detection systems. Our LIR based YVO_4_:Nd^3+^ thermometers had higher sensitivities (0.47–0.54% K^−1^) than other single Nd-doped materials. Despite the high thermal sensitivity and readout simplicity, it is worthy to note that LIR technique can be used only in the limited temperature range: 123–398 K. Thus, non-contact luminescence thermal sensor for higher temperature region should be based on line position or FWHM.

**Figure 5:**
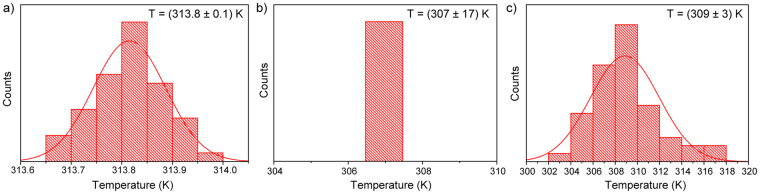
Thermal distributions calculated from (**a**) LIR, (**b**) line position and (**c**) FWHM of YVO_4_:Nd^3+^ 2.4 at.% NPs (T_heater_ = 313 K).

Along with sensitivity, an important parameter to describe the thermometric performance is the minimum temperature uncertainty (ΔT)^46^. This parameter defines the accuracy of temperature evaluation using nanothermometers and should be as small as possible. The minimum temperature uncertainty can be found with several methods performed and compared in our previous paper^34^. Here, 50 consecutive emission spectra of YVO_4_:Nd^3+^ 2.4 at.% NPs with fixed heating stage temperature (313 K) were measured. Then, the temperature corresponding to each spectrum was calculated using calibration curves based on various thermal sensing parameters: LIR, line position and FWHM. The obtained temperature distribution was fitted by Gauss function for each sensing parameter, and standard deviation was used as an estimation of the thermal resolution that can be achieved in a measurement with chosen method (Fig. 5). The best temperature uncertainty (ΔT = 0.1 K) was obtained for LIR method, thermal resolution of 3 K was obtained for FWHM technique, whereas we could not get ΔT based on line position, because all calculated temperatures had the same value. Therefore, in this case the temperature uncertainty was obtained from the spectral resolution. Single monochromator, which was used in our experimental setup, gave the spectral resolution of 0.0573 nm, which led to the very large uncertainty value of 17 K. The temperature uncertainty of line position method can be significantly improved by utilizing double or triple monochromator in the detection system.

## Summary

YVO_4_:Nd^3+^ NPs synthesized with modified Pechini method were successfully used as nanothermometers operating in the first and second biological windows. Luminescence thermal sensing was based on monitoring three different temperature dependent parameters: LIR, line position and FWHM. The highest thermal sensitivity was found for line position method reaching up to 0.75% K^−1^, whereas FWHM method demonstrated the lowest values. Despite high thermal sensitivity and readout simplicity, LIR technique can be used only in the limited temperature range (123–398 K) due to temperature-induced line broadening and shifting. Theoretical expressions and simplified functions were used to fit temperature behavior of both line position and FWHM. It was demonstrated that aforementioned luminescence temperature dependent parameters can provide thermal sensing in wide range (123–873 K), which is sufficient for majority of industry application. Doping concentration affects thermal sensitivity of LIR, line position and FWHM techniques similarly: the best values were obtained for sample with the highest luminescence intensity − YVO_4_:Nd^3+^ 2.4 at.%. The minimum temperature uncertainty (ΔT = 0.1 K) was obtained for LIR technique.

## Methods

YVO_4_:Nd^3+^ nanoparticles used in this work were synthesized using a previously reported modified Pechini method^47^. Fluorescence characterization was performed by using T64000 Raman Spectrometer. The Nd^3+^-doped YVO_4_ NPs were optically excited with a 808 nm single mode laser Coherent MBR-110 operating in CW mode. The laser beam was focused into the sample by using a 4x long working distance microscope objective (NA 0.1). The fluorescence was collected by using the same microscope objective and was spectrally analyzed by single spectrometer and liquid nitrogen cooled Symphony II and Peltier cooled Synapse CCD detectors. The temperature was controlled with a heating stage Linkam (Linkam THMS 600 of 0.1 °C temperature stability and 0.1 °C set point resolution).

## Electronic supplementary material

SUPPLEMENTARY INFO
