# Supplementary material for: YVO4:Nd3+ nanophosphors as NIR-to-NIR thermal sensors in wide temperature range
Source: Sci Rep. 2017 Dec 21;7:18002. doi: 10.1038/s41598-017-18295-w (PMC5740097; doi:10.1038/s41598-017-18295-w)
Supplement: Supplementary file 1 — SUPPLEMENTARY INFO [file 41598_2017_18295_MOESM1_ESM.pdf]

$YVO_4:Nd^{3+}$ nanophosphors as NIR-to-NIR thermal sensors in wide temperature range

I.E. Kolesnikov[*,1,2], A.A. Kalinichev[1], M.A. Kurochkin[1], E.V. Golyeva[3,4], E.Yu. Kolesnikov[5], A.V. Kurochkin[1], E. Lähderanta[2], M.D. Mikhailov[4]

[1] *St. Petersburg State University, 7/9 Universitetskaya nab., 199034, St. Petersburg, Russia*

[2] *Lappeenranta University of Technology LUT, Skinnarilankatu 34, 53850, Lappeenranta, Finland*

[3] *Peter the Great St. Petersburg Polytechnic University, St. Petersburg, Russia*

[4] *Scientific and Technological Institute of Optical Material Science, VNTs S. I. Vavilov State Optical Institute, Babushkina 36-1, 192171, St. Petersburg, Russia*

[5] *Volga State University of Technology, Lenin sqr. 3, 424000, Yoshkar-Ola, Russia*

One of the most important parameter for characterizing luminescent thermometers is the relative thermal sensitivity, which is defined as follows [S. A. Wade, S. F. Collins and G. W. Baxter, *J. Appl. Phys.*, 2003, **94**, 4743–4756]:

$$S = \frac{1}{\Lambda}\frac{\Delta\Lambda}{\Delta T}$$

where Λ is the temperature dependent parameter (LIR, line shift or FWHM) taken into account and ΔΛ expresses the change in this parameter with change of temperature, ΔT [Ł. Marciniak, A. Bednarkiewicz, D. Hreniak and W. Strek, *J. Mater. Chem. C,* 2016, **4**, 11284–11290].

The relative thermal sensitivity of all temperature dependent parameters was obtained at T=303 K (or at T=298 K in case of FWHM).

Here, we present the relative thermal sensitivity calculation based on LIR, line shift and FWHM for $YVO_4:Nd^{3+}$ 2.4 at.% NPs. Similar calculations have been also carried out for 0.6 and 4.8 at.%-doped samples.

LIR

$$S_{LIR} = \frac{1}{LIR_{303K}} \cdot \frac{|LIR_{323K} - LIR_{303K}|}{323-303} = \frac{1}{2.504} \cdot \frac{|2.236 - 2.504|}{323-303} = 0.0054\, K^{-1} = 0.54\,\%K^{-1}$$

Line shift

$$S_{\delta\text{v}} = \frac{1}{\delta\text{v}_{303K}} \cdot \frac{|\text{v}_{323K} - \text{v}_{303K}|}{323-303}$$

$$\delta\text{v}_{303K} = \text{v}_{0K} - \text{v}_{303K}$$

v_{0K} was calculated using parameters obtained from exponential fitting:

$$\text{v}_{0K} = 9445.8 - 39.6 = 9406.2\ \ cm^{-1}$$

$$\delta\text{v}_{303K} = \text{v}_{0K} - \text{v}_{303K} = 9406.2 - 9399.5 = 6.7\ \ cm^{-1}$$

So, the relative thermal sensitivity:

$$S_{\delta\text{v}} = \frac{1}{\delta\text{v}_{303K}} \cdot \frac{|\text{v}_{323K} - \text{v}_{303K}|}{323-303} = \frac{1}{6.7} \cdot \frac{|9398.5 - 9399.5|}{323-303} = 0.0075\, K^{-1} = 0.75\,\%K^{-1}$$

FWHM

$$S_{\Delta\text{v}} = \frac{1}{\Delta\text{v}_{298K}} \cdot \frac{|\Delta\text{v}_{323K} - \Delta\text{v}_{298K}|}{323-298} = \frac{1}{43.271} \cdot \frac{|44.84 - 43.271|}{323-298} = 0.0014\, K^{-1} = 0.14\,\%K^{-1}$$
